# Forced swim stress exacerbates inflammation-induced hyperalgesia and oxidative stress in the rat trigeminal ganglia

**DOI:** 10.3389/fpain.2024.1372942

**Published:** 2024-04-24

**Authors:** Jin Y. Ro, Youping Zhang, Jamila Asgar, Huizhong Shou, Man-Kyo Chung, Ohannes K. Melemedjian, Joyce T. Da Silva, Shou Chen

**Affiliations:** ^1^ Department of Neural and Pain Sciences, University of Maryland School of Dentistry, Baltimore, MD, United States; ^2^ Division of Biostatistics and Bioinformatics, Department of Epidemiology and Public Health, University of Maryland School of Medicine, Baltimore, MD, United States

**Keywords:** psychophysical stress, PCR array, peripheral inflammation, antioxidant, hyperalgesia

## Abstract

This study investigates the impact of combining psychophysical stress, induced by forced swim (FSS), with masseter inflammation on reactive oxygen species (ROS) production in trigeminal ganglia (TG), TRPA1 upregulation in TG, and mechanical hyperalgesia. In a rat model, we demonstrate that FSS potentiates and prolongs CFA-induced ROS upregulation within TG. The ROS levels in CFA combined with FSS group surpass those in the CFA-only group on days 4 and 28 post-treatment. FSS also enhances TRPA1 upregulation in TG, with prolonged expression compared to CFA alone. Furthermore, CFA-induced mechanical hyperalgesia is significantly prolonged by FSS, persisting up to day 28. PCR array analyses reveal distinct alterations in oxidative stress genes under CFA and CFA combined with FSS conditions, suggesting an intricate regulation of ROS within TG. Notably, genes like *Nox4*, *Hba1*, *Gpx3*, and *Duox1* exhibit significant changes, providing potential targets for managing oxidative stress and inflammatory pain. Western blot and immunohistochemistry confirm DUOX1 protein upregulation and localization in TG neurons, indicating a role in ROS generation under inflammatory and stress conditions. This study underscores the complex interplay between psychophysical stress, inflammation, and oxidative stress in the trigeminal system, offering insights into novel therapeutic targets for pain management.

## 1 Introduction

An increasing body of evidence suggests bidirectional relationships between stress and pain, manifesting in both acute and chronic contexts (1, 2). Repeated or prolonged exposure to physical or psychological stressors commonly induces physiological reactions that heighten the sensitivity to painful stimuli, a phenomenon referred to as stress-induced hyperalgesia (SIH). Most animal studies consistently demonstrate SIH across various types of stressors, among different rodent strains and species, and with diverse modalities of test stimuli [for review see (3)]. In humans, psychological stress has the potential to exacerbate chronic pain conditions (4, 5, 6), while prolonged pain can trigger maladaptive responses in the hypothalamic-pituitary-adrenal axis, the body's primary stress system (7, 8), establishing a vicious cycle where both factors amplify each other (9).

Several preclinical studies have explored the effects of psychophysical stress and pain processing in the orofacial system. Forced swim stress (FSS) enhances acute temporomandibular joint-evoked activity of nociceptive neurons in the medullary dorsal horn and enhances masseter muscle activities (10, 11). FSS, restrain stress as well as crowding stress aggravate pulpal nociception (12), and stress significantly increases dentin hypersensitivity in rats (13). Masseter muscle injury induces persistent visceral hypersensitivity for months, specifically in an estrogen-dependent manner, but only when the injury is combined with FSS (14). Furthermore, mice prone to succumb to social defeat stress exhibit heightened masseter muscle nociception, as evidenced by an increase in orofacial nocifensive behaviors and elevated c-Fos activity in the C1/C2 region following formalin injection (15). Nevertheless, the mechanisms underlying the heightened and intensified pain responses in the trigeminal system resulting from different types of psychophysical stress have not been thoroughly investigated.

Psychophysical stress has been identified as a catalyst for the generation of reactive oxygen species (ROS) in the brain (16–19), The resulting cellular and molecular changes, stemming from an imbalance in ROS metabolism, have been implicated in various CNS disorders, such as neurodegeneration, schizophrenia, anxiety, and depression, as outlined in the review by Salim (20). A recent study demonstrated that repeated and intermittent sound stress induces enduring non-inflammatory hyperalgesia in mice, accompanied by heightened oxidative stress and lipid oxidative damage, which could also be observed in fibromyalgia patient (21). Considering that injury and inflammation are recognized as contributors to ROS generation across nociceptive pathways, it is plausible to posit that psychophysical stress exacerbates oxidative stress, particularly under inflammatory conditions, thereby amplifying pathological pain responses. Despite this, limited information exists regarding psychophysical stress and the genes involved in ROS metabolism in nociceptive systems. In prior research, we demonstrated that masseter inflammation leads to the accumulation of ROS within the trigeminal ganglia (TG) (22). This increase in intraganglionic ROS was shown to contribute to inflammatory hyperalgesia through the regulation of TRPA1 expression and function within the TG. The objectives of this study are to investigate whether FFS (1) exacerbates inflammation-induced ROS production in the TG, (2) intensifies inflammatory hyperalgesia, (3) further upregulates TRA1, and (4) to conduct a PCR array study for a comprehensive evaluation of 94 genes related to oxidative stress in TG under inflammatory conditions, and to determine whether FSS alters the gene expression profiles.

## 2 Materials and methods

### 2.1 Animals

Adult male Sprague-Dawley rats, aged three to six months and weighing between 150 and 350 g, obtained from Harlan in Indiana, USA, were used in this study. These rats were kept in a room with controlled temperature and a 12-hour light-dark cycle, and they had unrestricted access to both food and water. All research procedures adhered to the guidelines outlined in the National Institutes of Health Guide for the Care and Use of Laboratory Animals (publication no. 80-23) and were conducted under the approval of the Institutional Animal Care and Use Committee at the University of Maryland Baltimore. We conducted the present study using only male rats to align with our previous studies (23, 24). However, we acknowledge that there may be potential differences in ROS accumulation between males and females that require further investigation.

### 2.2 Masseter inflammation

To induce inflammation, we administered a 50 μl injection of a solution containing 50% Complete Freund's Adjuvant (CFA) in isotonic saline (purchased from Sigma-Aldrich, St. Louis, MO) into the middle portion of the masseter muscle using a 27-gauge needle. For the injection, the rats were briefly placed under anesthesia with 3% isoflurane. The animals fully recover from the anesthesia within 5 min.

### 2.3 Forced swim

To investigate the impact of psychophysical stress on inflammatory conditions, we employed the repeated swim stress model, as it has previously demonstrated to increase muscle pain and cutaneous hyperalgesia (25). We adapted the procedures outlined in earlier studies (25, 26). Rats were individually housed and brought into a procedure room for three consecutive days, each day being subjected to swim stress once. On the first day, rats underwent forced swimming by placing them in a plastic cylinder (diameter: 30 cm, height: 50 cm) filled with 20 cm of water at a temperature of 24°C–26°C for a duration of 20 min. On the second and third days, the swim stress sessions were reduced to 10 min each. Throughout the swim sessions, the animals were continuously monitored. After each swimming session, the animals were towel-dried and allowed to dry in a warm environment (30 °C–33 °C) before being placed in the drying cage.

### 2.4 ROS assay in TG

The methods for the ROS assay were described in our previous studies (23, 24). Briefly, ROS levels were quantified using a cell-permeant oxidant-sensing probe 2’,7’-dichlorodihydrofluorescein diacetate (H_2_DCFDA; Invitrogen, Carlsbad, CA, USA). H_2_DCFDA is de-esterified within the cytoplasm and turns highly fluorescent upon oxidation. H_2_DCFDA detects hydrogen peroxide (H_2_O_2_), peroxyl radicals (ROO•), and peroxynitrite (ONOO−), but it is possible that other biologically relevant ROS, such as superoxide radicals (O2•−) and hydroxyl radicals (OH•), are also involved. In the CFA groups, rats received an injection of CFA into the left masseter muscle (4–6 rats per group). In the CFA with FS groups, rats underwent three daily sessions of swim stress one day after the CFA injection into the masseter muscle (4–6 rats per group). The ipsilateral TG to the injected muscle was removed either 4, 7, 14, or 28 days after the CFA injection. A separate group of rats that received a vehicle injection in the masseter muscle served as a control group for both the CFA-only and CFA with FS groups (4–8 rats per group). TG was quickly removed and washed with phosphate-buffered saline (PBS). Immediately after extraction and dissection, the tissues were minced finely in PBS and were incubated in 96-well plates in 200 µl PBS for 30 min at 37°C. The background fluorescence for each specimen was determined with a fluorimeter (DTX880 Multimode Detector, Beckman Coulter) at 485 nm for excitation and 535 nm for emission. After the background reading, H_2_DCFDA was added to each well to a final concentration of 10 µM. The plates were again incubated for 30 min at 37°C, and the fluorescence was re-measured. ROS levels were estimated as the intensity of fluorescence after subtraction of the background fluorescence (Multimode Analysis Software). The results from CFA- or vehicle-treated group were normalized to the results from naïve rats that did not receive either CFA or vehicle.

### 2.5 Assessment of masseter mechanical hyperalgesia

To assess persistent mechanical hyperalgesia in the masseter muscle under both inflammatory conditions with and without swim stress, we employed a rodent behavioral model developed for evaluating masseter sensitivity in awake rats (27). Detailed procedures have been extensively described in our previous studies (28, 29). In brief, the rats were acclimated to stand on their hind paws and lean against the experimenter's hand, which was protected by a leather work-glove. The rats were not physically restrained but maintained this position long enough for the experimenter to apply Von Frey filaments to the skin covering the masseter muscle. An ascending series of Von Frey monofilaments (Stoelting, Wood Dale, IL., USA) was employed. Each filament was tested five times with a few seconds between each test. The response threshold was defined as the lowest force of the filaments that elicited at least three active head withdrawal responses out of five tests. Response frequencies were calculated as [(number of responses/number of stimuli) × 100%] for a range of filament forces. Subsequently, a non-linear regression analysis was conducted to determine the EF50 value, representing the filament force (in grams) required to produce a 50% response frequency. The EF50 value served as a measure of mechanical threshold, with a decrease indicating mechanical hyperalgesia. The changes in mechanical thresholds were compared between CFA only group with CFA and FS treated groups (*n* = 8 per group).

### 2.6 PCR array

TG samples collected from naïve rats and rats in CFA with and without FS groups at various time points were prepared for the Rat Oxidative Stress RT2 Profiler PCR Array (Rotor-Gene® Format, Cat. no. 330231 PARN-065ZR, Qiagen, Aarhus, Denmark) following the manufacturer's instructions. The PCR Array kit contained primers for 94 gene transcripts related to oxidative stress, including peroxidases, and genes involved in ROS metabolism. The results were analyzed by the Boston University Analytical Instrumentation Core (Boston, MA, USA.) To determine the expression profiles of genes that may regulate intraganglionic ROS levels, we collected TG from rats treated with CFA, both with and without FS, and conducted the PCR array assay (triplicates from 3 rats per group). TG samples prepared on days 4, 7, 14 after CFA treatment and days 4, 7, and 14 after CFA and FS combined treatment. The expression levels of the 94 genes at each time point were compared to those of TG samples prepared from the rats treated with a vehicle.

### 2.7 Real-time RT-PCR

Total RNA was extracted from dissected TG ipsilateral to the inflammation using a RNeasy kit (Qiagen Sciences, Germantown, MD) followed by DNase treatment to remove genomic DNA. Reverse transcription was carried out using SuperScript II kit (Invitrogen, Waltham, MA) was used to generate cDNA from 500 ng of RNA along with 2.5 ng of random primer per reaction. Real-time PCR analysis of cDNA (equal to 15 ng of RNA) was performed using Maxima SYBR Green/ROX qPCR Master Mix in an Eppendorf Mastercycler Ep Realplex 2.0 (Fermentas, Forest City, CA, USA). In all our RT-PCR experiments, each sample was analyzed in triplicates, and we routinely added a control with no template as a means of checking for any nucleic acid contamination, and a control with no reverse transcriptase to verify that there was no DNA contamination in the RNA preparation. The no template control also serves to identify any potential formation of primer dimers during the SYBR Green assay. The following primer pairs were used to detect *Trpa1* mRNA: forward 5’-TCCTATACTG GAAGCAGCGA-3’, reverse 5’-CTCCTGATTGCCATC GACT-3’, *Duox1* mRNA: forward 5’-TGTGCAAGATTTTTGGCCCG-3’, reverse 5’-CGAGAGTGCAGGGTTGATGT-3’, and GAPDH, mRNA, used as a control: forward 5’-TCACCACCAT GGAGAAGGC G-3’, reverse 5’-GCTAAGCAGTTGGTG GTGCA-3’. We obtained the ratios between *Trpa1* and GAPDH and *Duox1* and GAPDH to calculate the relative abundance of mRNA levels in each sample. Relative quantification of the *Trpa1 and Duox1* mRNA was calculated by the comparative CT method (2^−ΔΔCT^ method) between control and experimental groups. The relative fold changes were compared between naïve and CFA-treated rats (4–5 per group) over the course of 14 days, and between naïve rats and those treated with both CFA and FS (4–6 per group) over the course of 28 days.

### 2.8 Immunohistochemistry (IHC)

The rats were transcardially perfused with cold phosphate buffered saline (PBS), followed by 4% paraformaldehyde in PBS (250 ml, pH 7.3–7.4; Sigma, St. Louis, MO, USA). TG were extracted and post-fixed for 90 min, placed in 30% sucrose solution at 4°C overnight and sectioned coronally at 12 μm. Every eighth section was collected and mounted on gelatin-coated slides for double-labelling immunohistochemistry. After blocking, the sections were double-labeled overnight at room temperature with primary antibodies: mouse anti DUOX1 (1:200, sc-393096, Santa Cruz Biotechnology, Inc), guinea pig anti NeuN (1;200, 266014, Synaptic Systems), a specific marker for neurons, or rabbit anti DUOX1 (1:200, PA585452, Invitrogen), mouse anti GFAP (1:200, G3893, Sigma), an antibody directed to glial fibrillary acid protein, a marker for satellite glia to determine localization of DUOX1 in different cell types. For immunofluorescence, sections were incubated for 1 h in Cy3-conjugated goat anti-mouse (1:500, 115-165-166, Jackson ImmunoResearch), Alexa 488-conjugated goat anti-guinea pig (1:500; A11073, Invitrogen,), or Cy3 -conjugated goat anti rabbit anti (1:500; 115-165-003, Jackson ImmunoResearch), Alexa 488-conjugated goat anti-mouse (1:500; A11001, Invitrogen) at room temperature.

### 2.9 Western blotting

Total proteins were extracted from the TG of naïve and experimental rats (5 rats per group). The protein samples were dissolved in RIPA buffer containing protease inhibitor cocktail. The protein concentration of lysates was determined using Bio-Rad protein assay kit (Bio-Rad, Hercules, CA, USA). Fifty micrograms of protein for each sample were separated on 4%–12% NuPAGE gel with MOPS SDS running buffer and transferred to a PVDF membrane (Bio-rad, Hercules, CA, USA). After blocking for 1 h in 5% milk PBST at room temperature, membranes were probed with primary antibodies for TRPA1 (1:1,000, Millipore #ABN-1009, Burlington, MA), DUOX1 (1:500, Invitrogen, PA5-85452, Waltham, MA) and an internal control protein β actin (1:20,000, Millipore, #A1978, Burlington, MA), diluted in blocking solution. The TRPA1 antibody was raised against the N-terminus of rat TRPA1 and detects a 90–98-kDa protein, which disappears in TG lysates probed with TRPA1 antibody pre-incubated with a commercially available peptide used to generate the antibody. We have validated the specificity of this antibody in our previous study (30). Membranes from TG samples were incubated with primary antibodies overnight at 4°C and washed four times with PBST. HRP-conjugated secondary antibodies (anti-rabbit secondary antibody (Cell Signaling, Danvers, MA) and anti- mouse secondary antibody (Millipore, Burlington, MA) were diluted to 1:5000 in PBST and incubated with membranes for 1 h at room temperature. Bands were visualized using ECL (Western Lightning, PerkinElmer Inc., Waltham, MA, USA) or ECL plus Western blotting detection reagent (Lumigen PS-3, GE Healthcare, Chicago, IL). Protein level for TRPA1 was normalized to that of GAPDH within the same sample.

### 2.10 Enzyme-linked immunosorbent assay (ELISA)

Blood samples were obtained from the artery on the ventral aspect of the rat's tail both at baseline and the next day upon completion of the exercise regimen. The rats were anesthetized with isoflurane (1.5%–2%) for all blood collection procedures. These blood samples were collected between 12 pm and 3 pm, subsequently centrifuged to separate the serum, and then stored at −20 °C until the assay day. The concentrations of corticosterone (ng/ml) levels from the serum samples were evaluated using ELISA assay kits provided by Cayman Chemical Company, following the manufacturer's instructions.

### 2.11 Statistical analyses

The time-dependent changes in mechanical hyperalgesia before and after CFA or CFA with FS were analyzed with a Two-Way analysis of variance (ANOVA) with repeated measures. Data obtained from RT-PCR experiments were analyzed with a one-way ANOVA on means or Kruskal–Wallis one-way ANOVA on ranks depending on the outcome of a normality test. Unless otherwise indicated, statistical comparisons of two independent groups were made with either Student's *t*-test or Mann–Whitney Rank Sum test. Data are presented as mean ± SE and differences were considered significant at *p* < 0.05. All multiple group comparisons were followed by Bonferroni *post hoc* test. False discovery rate (FDR) was used to correct for multiple comparisons with an adjusted cut-off of 0.05. We used G*Power Software (Heinrich-Heine, Universität Düsseldorf) to perform power analysis which confirms that the sample sizes we used yielded a power greater than 0.85 with a moderate effect size of Cohen's d = 0.5.

## 3 Results

### 3.1 FSS potentiates and prolongs CFA-induced ROS upregulation within TG

**Figure 1:**
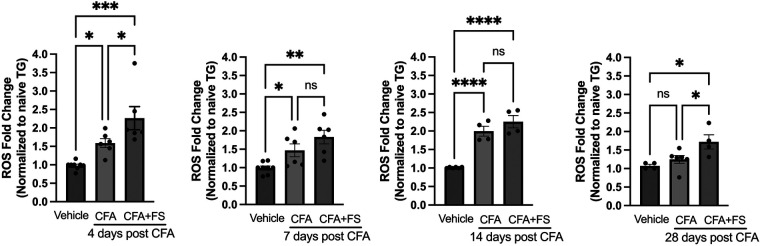
Forced swim stress augments CFA-induced elevation of ROS upregulation in TG. Changes in ROS within TG following masseter inflammation were assessed by measuring relative intensity of fluorescence using H2DCFDA, an indicator for ROS, of TG obtained from naive, CFA, CFA combined with forced swim (FS) stress or vehicle treated rats on days 4, 7, 14 and 28 post CFA treatment. We used 5–8 naïve rats for normalization for each time point. Student *t*-test was used for statistical analysis at each time point. *p < 0.05, **p < 0.005, ***p < 0.0005, and ****p < 0.00005 for significant differences between groups. NS = not significant. Data are presented as the mean ± SEM.

In our previous study, we observed a significant increase in ROS levels in TG starting one day after inducing masseter inflammation with CFA. This elevated ROS level persisted for up to 14 days (24). In the current study, we investigated whether combining inflammation with FSS would further elevate ROS levels in the TG. We compared ROS production in the TG of rats treated with CFA, with or without FSS, to that of rats treated with a vehicle. Assessments were made on days 4, 7, 14, and 28 following the injection of CFA or the vehicle into the masseter muscle. Fluorescent signals from the TG of both CFA-treated rats, with or without FSS, were significantly higher than those from vehicle-treated rats on days 4, 7, and 14 after CFA treatment (Figure 1). ROS levels were consistently higher in the CFA combined with FSS group compared to the CFA-only group at all time points, although the statistical significance was only reached on days 4 and 28 (*p* < 0.05 for days 4 and 28 post-CFA vs. CFA + FSS). On day 28, the ROS level had returned to baseline for the CFA-only group, while it remained significantly elevated in the CFA combined with FSS group, surpassing both the vehicle and CFA-only groups (*p* < 0.05 for Vehicle and CFA + FSS vs. CFA). These results indicate that FSS significantly enhances inflammation-induced upregulation of ROS production in the TG and prolongs the duration of ROS production.

### 3.2 FSS potentiates TRPA1 upregulation within TG and prolongs CFA-induced mechanical hyperalgesia

**Figure 2:**
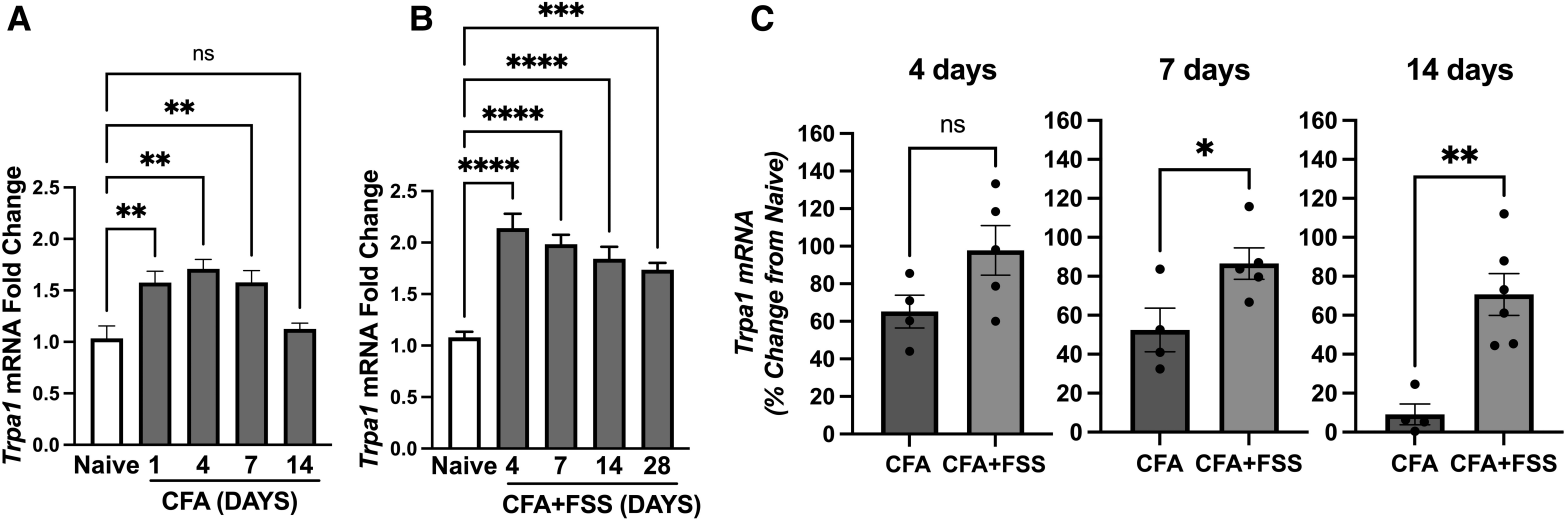
Forced swim stress further increases CFA-induced upregulation of *Trap1* mRNA expression in TG. Real time RT-PCR data showing (**A**) CFA- and (**B**) CFA-combined with forced swim stress-induced changes in *Trpa1* mRNA levels in TG compared to that of naïve untreated TG. (**C**) Bar graphs compare percent changes in mRNA expression levels from naïve between the CFA only and CFA combined with forced swim stress groups for the comparable days following CFA treatment. *p < 0.05, **p < 0.005, ***p < 0.0005, and ****p < 0.00005 for significant differences between groups. NS = not significant. Data are presented as the mean ± SEM.

Since intraganglionic ROS leads to upregulation of *Trpa1* expression in TG (24), we investigated whether the increased production of ROS under the FSS condition leads to corresponding changes in the expression of *Trpa1* in the TG. Figure 2A shows that CFA treatment in the masseter muscle leads to a substantial upregulation of *Trpa1* within the first 7 days after CFA treatment. When CFA treatment was combined with FSS, *Trpa1* expression within the TG remained significantly elevated for up to 28 days after CFA treatment (Figure 2B). Since direct comparisons between the two groups were not feasible, we calculated the percent changes in fold changes of *Trpa1* relative to the expression level of naïve rats under each condition and compared between the comparable days following CFA treatment, i.e., CFA 4, 7, and 14 days vs. CFA + FSS 4, 7, 14 days, respectively. FSS led to a higher level of percent change in *Trpa1* expression at all time points, with a statistically significant increase on days 7 and 14 compared to that observed under CFA treatment alone (Figure 2C).

**Figure 3:**
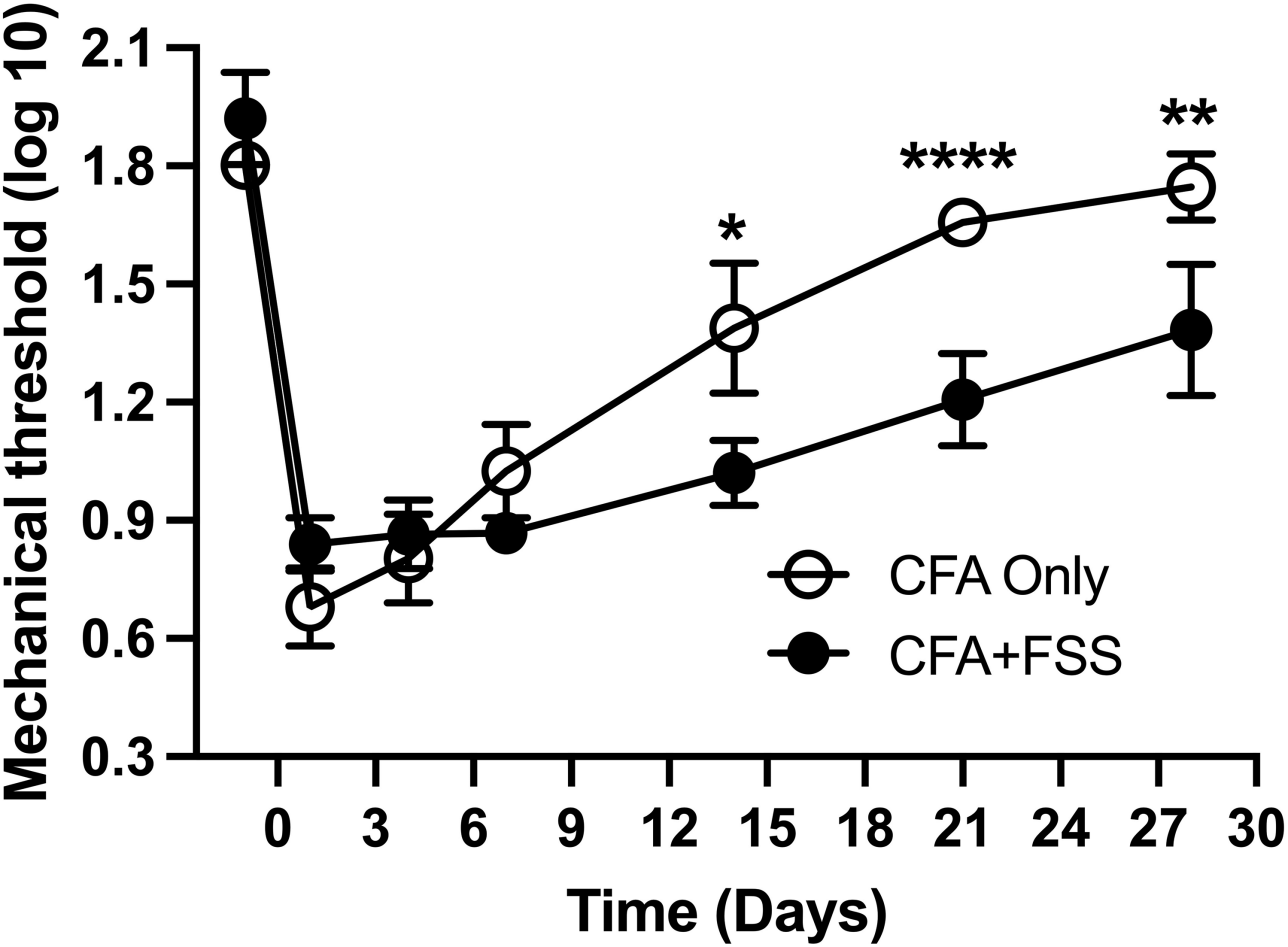
Forced swim stress exacerbates CFA-induced mechanical hyperalgesia. The line graph illustrates changes in mechanical hyperalgesia in rats treated with complete Freund's adjuvant (CFA) in the masseter muscle, comparing them to rats treated with CFA in conjunction with forced swim (FS) stress for 3 days. The mechanical force (g) required to elicit head withdrawal responses in 50% of the trials was log-transformed and plotted for the pre-treatment period and days 1, 4, 7, 14, 21, and 28 after CFA treatment. Two-way ANOVA with repeated measures was employed for statistical analysis. *p < 0.05, **p < 0.01, and ****p < 0.0001 denote significant differences between the CFA group and the combined CFA and forced swim stress group (*n* = 8 per group).

We then investigated whether the increased levels of intraganglionic ROS and *Trpa1* expression alter pain-related responses by evaluating the magnitude and time course of CFA-induced mechanical hyperalgesia in two groups of rats: those treated exclusively with CFA and those subjected to a combination of CFA treatment and FSS. We have confirmed that CFA treatment in the masseter muscle induces significant mechanical hyperalgesia, with the peak occurring in the first day, maintained for 7 days, and gradually returning to baseline within our observation period (Figure 3). Rats subjected to both CFA and FSS displayed a similar magnitude of mechanical hyperalgesia during the first week after CFA treatment. However, they exhibited a greater degree of hyperalgesia compared to the rats treated with CFA alone in the subsequent weeks, and this significant hyperalgesia was still evident on day 28. These findings indicate that FSS significantly prolongs CFA-induced mechanical hyperalgesia. We did not include an FSS-alone group in our study since FSS alone does not cause significant alterations in masseter mechanical thresholds (14).

### 3.3 PCR array analyses on oxidative stress genes

Table 1 displays the genes exhibiting significant alterations in fold regulations under both CFA-only and CFA combined with FSS conditions across all time points, relative to the control condition. Notably, there were fewer genes with increased fold regulations compared to those with decreased fold regulations under both conditions at all time points. Furthermore, the overall count of genes displaying significant fold regulations was greater under the combined CFA and FSS condition than under the CFA-only condition at all time points, particularly at the 7-day post-CFA treatment interval. Additionally, the FSS condition was linked to a more substantial magnitude of changes in fold regulations.

**Table 1:** All genes showing significant changes compared to the control condition (corrected for multiple *t*-tests).

CFA 4d		CFA + FS 4d		CFA 7d		CFA + FS 7d		CFA 14d		CFA + FS 14d	
*Apc 1.3*	*Hba1 −5.6*	*Duox1 1.7*	*Nox4 −14.8*	*Sqstm1 1.3*	*Hba1 −4.3*	*Ift172 1.5*	*Nox4 −21.5*	*Sqstm1 1.4*	*Nox4 −14.4*	*Duox1 1.7*	*Nox4 −17.0*
*Gpx2 1.4*	*Cybb −2.0*	*Apc 1.4*	*Hba1 −7.2*	* *	*Slc38a5 −3.0*	*Apc 1.4*	*Hba1 −11.0*	*Apc 1.3*	*Hba1 −3.9*	*Sqstm1 1.4*	*Hba1 −10.7*
* *	*Ccl5 −1.9*	*Ift172 1.4*	*Gpx3 −3.1*	* *	*Sod3 −1.1*	*Duox2 1.4*	*Epx −5.7*	*Dnm2 1.3*	*Gpx3 −2.7*	*Apc 1.3*	*Gpx3 −3.4*
* *	*Ncf1 −1.9*	*Dnm2 1.3*	*Cybb −1.9*	* *	* *	*Sqstm1 1.4*	*Gpx3 −3.1*	*Gclc 1.3*	*Ccl5 −1.8*	*Ift172 1.3*	*Slc38a5 −2.4*
* *	*Ptgs2 −1.9*	*Duox2 1.3*	*Rtc −1.8*	* *	* *	*Txnrd1 1.4*	*Cybb −2.3*	*If172 1.3*	*Ptgs2 −1.8*	*Als2 1.2*	*Cybb −2.2*
* *	*Txnip −1.5*	*Gclc 1.3*	*Ccl5 −1.7*	* *	* *	*Als2 1.3*	*Ncf1 −1.8*	*Apoe 1.2*	*Rtc −1.7*	*Slc38a1 1.2*	*Ptgs2 −2.1*
* *	*Rtc −1.4*	*Slc38a1 1.3*	*Cyba −1.5*	* *	* *	*Apoe 1.3*	*Ccl5 −1.7*	*Hprt1 1.2*	*Cyba −1.5*	*Txnrd2 1.2*	*Ncf1 −1.9*
* *	*Prdx4 −1.3*	*Sqstm1.3*	*Ncf1 −1.5*	* *	* *	*Ctsb 1.3*	*Slc38a5 −1.7*	*Slc38a1 1.2*	*Cygb −1.5*	*Dnm1l 1.2*	*Cyba −1.8*
* *	* *	*Txnrd1 1.3*	*Ncf2 −1.5*	* *	* *	*Dnm2 1.3*	*Cyba −1.6*	*Txnrd1 1.2*	*Ehd2 −1.3*	* *	*Cygb −1.5*
* *	* *	*Apoe 1.2*	*Gpx1 −1.4*	* *	* *	*Gclc 1.3*	*Ptgs2 −1.6*	* *	*Sod3 −1.3*	* *	*Gpx1 −1.4*
* *	* *	*Cat 1.2*	*Lpo −1.4*	* *	* *	*Slc38a1 1.3*	*Ncf2 −1.5*	* *	*Txnip −1.3*	* *	*Ncf2 −1.4*
* *	* *	*Sod2 1.1*	*Sod3 −1.4*	* *	* *	*Cat 1.2*	*Gpx1 −1.4*	* *	*Actb −1.2*	* *	*Rtc −1.4*
* *	* *	* *	*Gpx7 −1.3*	* *	* *	*Hprt1 1.2*	*RGDC −1.4*	* *	*Prdx4 −1.2*	* *	*Hmox1 −1.3*
* *	* *	* *	*Prdx4 −1.3*	* *	* *	*Dnm1l 1.2*	*Cygb −1.3*	* *	*Vim −1.2*	* *	*Sod3 −1.3*
* *	* *	* *	*Actb −1.2*	* *	* *	*Prnp 1.2*	*Ehd2 −1.3*	* *	* *	* *	*Ccs −1.2*
* *	* *	* *	* *	* *	* *	*Prdx5 1.1*	*Prdx4 −1.3*	* *	* *	* *	*Txnip −1.2*
* *	* *	* *	* *	* *	* *	*Sod2 1.1*	*Sod3 −1.3*	* *	* *	* *	*Actb −1.2*
* *	* *	* *	* *	* *	* *	* *	*Hmox1 −1.2*	* *	* *	* *	* *
* *	* *	* *	* *	* *	* *	* *	*Txnip −1.2*	* *	* *	* *	* *
* *	* *	* *	* *	* *	* *	* *	*Actb −1.2*	* *	* *	* *	* *
* *	* *	* *	* *	* *	* *	* *	*Prdx1 −1.1*	* *	* *	* *	* *

**Figure 4:**
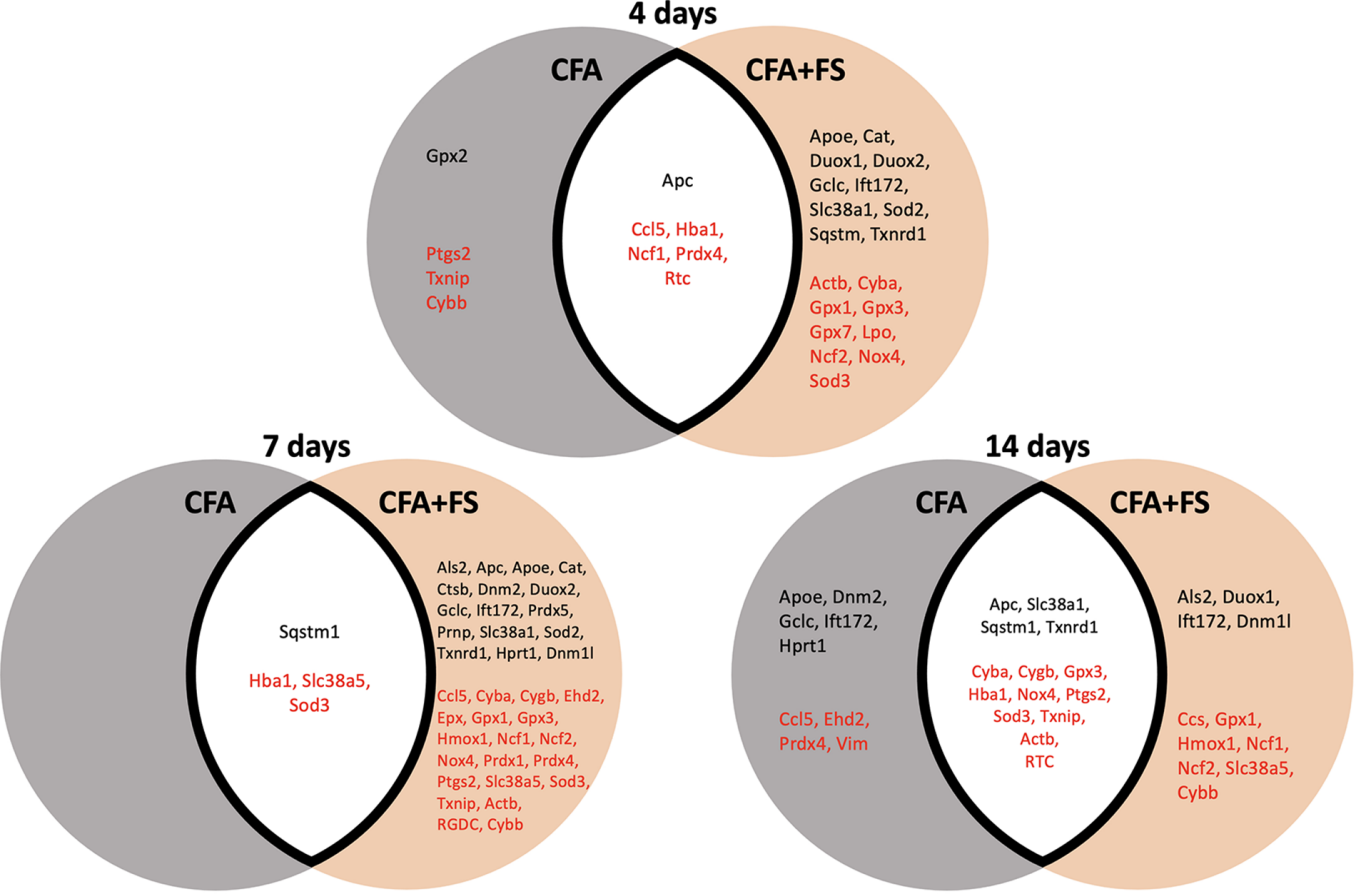
Venn diagrams illustrate genes with significant changes in fold regulations under two conditions: CFA alone and CFA combined with forced swim stress, at different time points. The grey circle represents the CFA-only condition, while the brown circle represents the CFA and stress combined condition. Genes in the white space exhibit significant changes under both conditions. Genes in black font signify upregulation, whereas genes in red font indicate downregulation.

To identify genes specifically linked to inflammatory responses and those affected by combined conditions, we segregated genes exhibiting significant fold regulations based on experimental conditions (Figure 4). Only four genes were identified for the CFA condition alone at 4 days, and nine genes at 14 days post-CFA treatment. No gene was identified for the CFA-only condition at the 4-day mark. In contrast, a considerable number of genes displayed significant alterations when CFA was combined with FSS, with the most pronounced effect observed at the 7-day time point.

For all genes that were evaluated, we selected genes that showed significant changes in fold regulation under at least two conditions. Of these, we identified 11 genes that exhibited greater than ±1.5 changes in fold regulations (Table 2). *Nox4* exhibited the most substantial decrease in fold regulation, with a greater magnitude of reduction observed under the FSS condition. *Gpx3* and *Cyba* displayed a similar pattern of alterations in gene expression, albeit with smaller decreases in fold regulations. *Hba1* underwent significant downregulation across all conditions, with more pronounced reductions in fold regulations under FS conditions. The expression levels of *Cybb* and *Ncf1* were predominantly reduced under FS conditions. *Ccl5*, *Slc38a5*, and *Rtc* exhibited sporadic changes under two or more conditions. Notably, *Duox1* was the sole gene to demonstrate a noteworthy increase in fold regulations, particularly during FS conditions. Our separate analysis of the identical set of genes one day after CFA treatment also uncovered an elevation of 1.8-fold regulations for *Duox1* (data not shown).

**Table 2:** Genes that exhibited greater than ± 1.5 changes in fold regulations.

* *	*Nox4*	*Hba1*	*Gpx3*	*Slc38a5*	*Cybb*	*Ptgs2*	*Ccl5*	*Ncf1*	*Cyba*	*Rtc*	*Duox1*
CFA 4d		−5.6			−2.0	−1.9	−1.9	−1.9			
CFA 7d		−4.3		−3.0							
CFA 14d	−14.4	−3.9	−2.7			−1.8	−1.8		−1.5	−1.7	
CFA + FS 4d	−14.8	−7.2	−3.1		−1.9			−1.5	−1.5	−1.8	1.7
CFA + FS 7d	−21.5	−11	−3.1	−1.7	−2.3	−1.6	−1.7	−1.8	−1.6		
CFA + FS 14d	−17.0	−10.7	−3.4	−2.4	−2.2	−2.1		−1.9	−1.8		1.7

### 3.4 Validation of *Duox1* expression in TG

**Figure 5:**
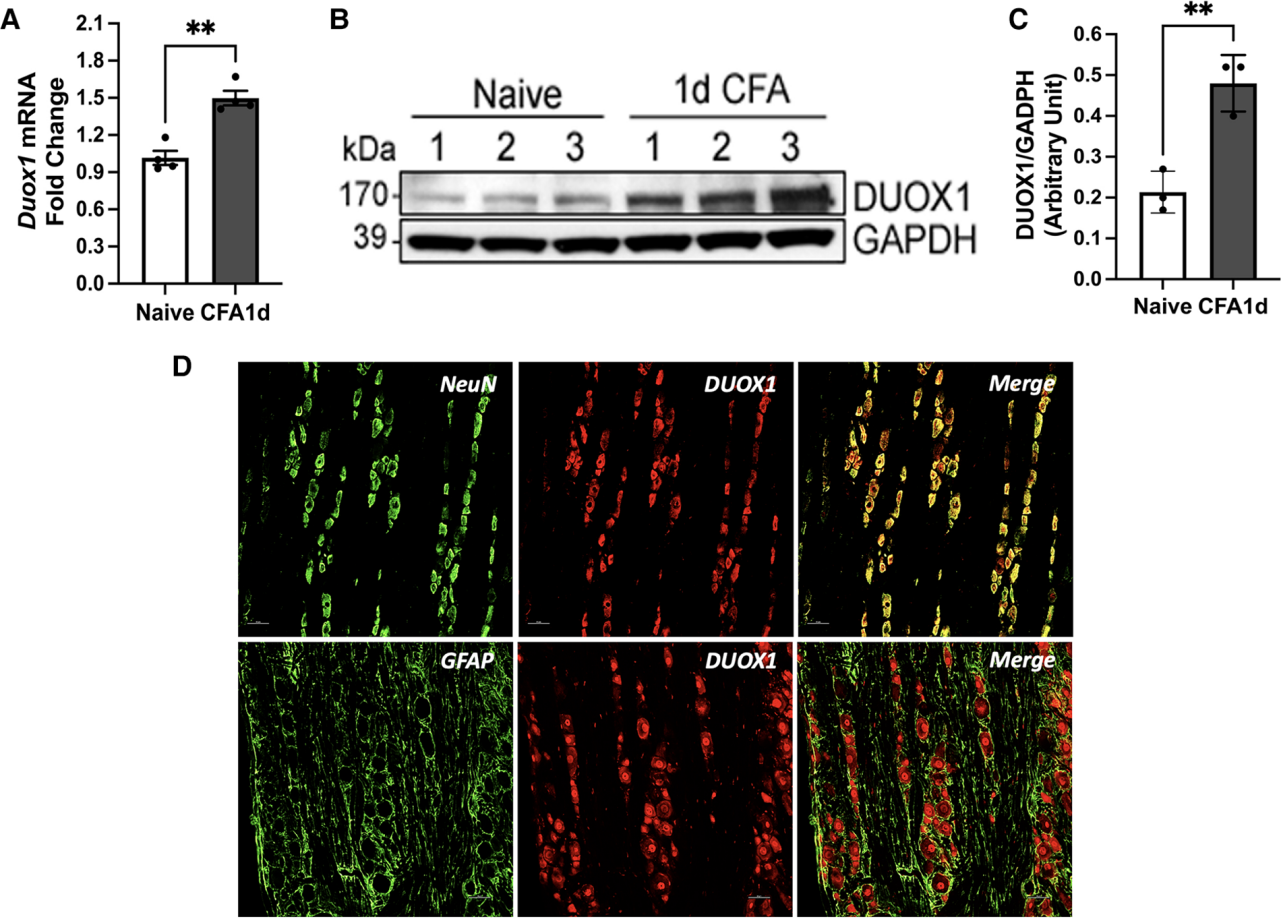
*Duox1* mRNA and DUOX1 protein expression TG. (**A**) RT-PCR analysis of Duox1 from TG of naive and CFA-inflamed rats. In CFA-treated rats, TG was analyzed one day after CFA administration in the masseter muscle. **p < 0.05. Each group consisted of 4 animals, and data are shown as mean ± S.E.M (**B**) Immunoblots of DUOX1 and GAPDH in naïve and CFA-treated TG. (**C**) Averaged relative optical density (DUOX1/GAPDH) between naïve untreated and CFA-treated TG samples. **p < 0.05. Each group consisted of three animals, and data are shown as mean ± S.E.M (**D**) The top row displays double labeling of DUOX1 with NeuN (a neuronal marker). DUOX1-positive elements were mainly detected in the cytoplasm of TG neurons. The bottom row shows double labeling with DUOX1 and GFAP (a marker for satellite glia). The neuropil stained positive for GFAP did not overlap with DUOX staining. Scale bar 25 μm.

To validate our PCR array results, we conducted RT-PCR and western blot analysis to confirm the expression of *Duox1* in TG. We selected *Duox1* because it was the only gene that displayed significant upregulation under both CFA and FSS conditions. Our RT-PCR experiment confirmed the expression of *Duox1* mRNA in the TG, independently corroborating the PCR array results. Furthermore, we observed a significant increase in its expression level one day after the CFA treatment. (Figure 5A) In line with the RT-PCR data, western blot experiments showed that the DUOX1 protein in TG exhibited a significant upregulation at 1 day after CFA treatment compared to TG from naïve rats (Figures 4B,C). We then conducted immunohistochemistry (IHC) study to demonstrate the localization of DUOX1 in TG. Somatic labelling of DUOX1 could be clearly detected in TG. Our double labeling experiments confirmed with NeuN that DUOX1 immunoreactivity observed in TG was neuronal. DUOX1 immunoreactivity did not co-localized with GFAP stained elements, suggesting that DUOX1 is not expressed in satellite glial cells. These IHC observations were confirmed in three independent experiments. Our western blot and IHC experiments constitute the first evidence of DUOX1 expression in somatic ganglia. The observed changes in Duox1 mRNA, as detected with the PCR array, were accompanied by corresponding changes in the protein level, suggesting potential involvement of Duox1 under inflammatory and stress conditions. Nevertheless, further biochemical validation and functional assays are necessary to fully understand the significance of Duox1 expression under inflammatory conditions with and without stress.

### 3.5 Effects of forced swim stress alone

**Figure 6:**
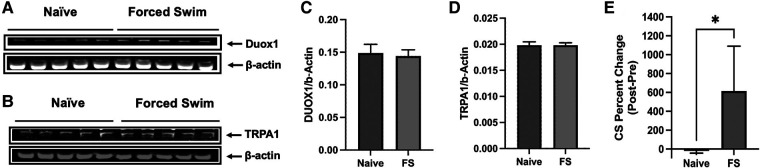
Effects of FSS only. (**A,B**) Immunoblots of DUOX1 and β-actin and TRPA1 and β-actin from naïve and FSS treated rats. (**C,D**) Averaged relative optical density (DUOX1/β-actin and TRPA1/β-actin) from naïve and FSS treated rats. Each group consisted of five animals, and data are shown as mean ± S.E.M. (**E**) Changes in blood corticosterone levels in naïve and rats under FS stress were compared before and after the treatment. Blood samples from naïve rats were drawn at the same time for FS stress rats. Graphs represent percent changes between the two time points (mean ± SEM). *p < 0.05 (Mann-Whiney test).

In our study we did not include the FSS only group since the identical protocol of FSS as we utilized does not induce heightened sensitivity in the masseter muscle (14). Also, the primary aim of the study was to explore the impact of stress on oxidative stress within TG in the context of existing inflammation or injury, building upon our prior findings (24). In order to further demonstrate that FSS alone does not have significant impact in TG, we assessed the TRPA1 and DUOX1 protein levels. Our results showed that FSS alone does not alter the expression levels of TRPA1 or DUOX1 in the TG (Figures 6A–D), which aligns with findings on behavioral responses (14). These experiments provided additional information suggesting that FS alone does not result in a significant elevation of ROS, thus not affecting masseter hypersensitivity or TRPA1 expression. The FSS model we utilized in the current study is a well-established protocol with ample data demonstrating the induction of stress in animals. Forced swimming sessions ranging from 1 to 7 days daily have been shown to lead to a dramatic increase in plasma corticosterone (CS) levels, indicating elevated stress responses in rats (31–33). More recently, Dong et al. (2016) reported that immobility time, which is used as an index of stress, during the first 5 min of each FSS increased daily (34). We also conducted our own ELISA assay to measure blood corticosterone levels before and after the FSS. Our data confirmed the increased levels of CS, demonstrating that the rats were indeed stressed following 3 days of forced swim (Figure 6E).

## 4 Discussion

Chronic musculoskeletal pain is linked to elevated stress levels (35), and stress is proposed to play a pivotal role in the progression from acute to chronic musculoskeletal pain (36–39). FSS has been demonstrated to induce musculoskeletal hyperalgesia in rats, a response effectively inhibited by milnacipran, a dual serotonin/norepinephrine uptake inhibitor. This suggests a potential involvement of central norepinephrine and/or serotonin in the stress-induced enhancement of muscle nociception (25) FFS also induces identical musculoskeletal hyperalgesia in mice. In this study, corticotropin-releasing factor receptors in the spinal cord area play a crucial role in the development of stress-induced musculoskeletal hyperalgesia (40). Within the trigeminal system, psychological stress amplifies mechanical sensitivity in both temporal and masseter muscles bilaterally. This heightened sensitivity can be mitigated by diazepam, an allosteric modulator of GABAa receptors (41). These findings align with studies demonstrating that chronic exposure to various forms of psychophysical or psychological stress induces hyperalgesia in naïve animals (26, 42, 43). However, the FSS paradigm, identical to the one employed in this study, failed to induce significant hyperalgesia in the masseter muscle in non-inflamed rats (14). This implies that variations in procedures and methodologies may impact the expression of stress-induced pain responses.

In addition to inducing non-inflammatory hyperalgesia in naïve animals, psychophysical stress amplifies hyperalgesic responses in animals experiencing inflammatory conditions (44–48). The exacerbation of inflammatory hyperalgesia through chronic and repeated sound stress involves contributions from both the sympathoadrenal (epinephrine) and the hypothalamic-pituitary adrenal (corticosterone) neuroendocrine stress axes (46, 47). The contribution of nociceptors in stress-induced exacerbation of inflammatory hyperalgesia is supported by a recent study (48), which demonstrated that cellular pathways involving miR-3120 regulation on Hsc70, leading to the overexpression of TRPV1 in DRG neurons, mediate FSS-induced mechanical hyperalgesia under inflammatory conditions. FSS also enhances inflammatory responses arising from the masseter muscle (14, 49). Our data, demonstrating that FSS results in prolonged mechanical hyperalgesia in the masseter muscle when combined with inflammation, contribute to these observations. However, the specific mechanism(s) leading to stress-induced exacerbation of inflammatory hyperalgesia in the orofacial muscles remain unclear.

In our prior research (24), we established that inflammation in the masseter muscle induced by CFA results in the prolonged presence of ROS in the TG. These TG-confined ROS play a significant role in contributing to inflammatory pain responses by causing a sustained upregulation of the pronociceptive gene, *Trpa1*. ROS accumulated within TG participate in the pathogenesis of inflammatory pain by directly activating multiple types of transient receptor potential (TRP) channels, including TRPA1 and TRPV1 (50). ROS can also indirectly activate TRPA1 through oxidative aldehydes, such as 4-hydroxy-2E-nonenal (4-HNE), mediating inflammatory and neuropathic pain (51, 52). Furthermore, the accumulation of ROS in the TG generates inflammatory cytokines and chemokines via transient receptor potential melastatin 2 (TRPM2) channels, which, in turn, promote nociceptor sensitization by increasing TRPA1 expression (23). Importantly, our current data show that FSS-induced exacerbation of inflammatory hyperalgesia is accompanied by a prolonged increase in TRPA1 expression and ROS accumulation within the TG. Although we did not functionally examine whether the augmented ROS are directly responsible for the exacerbation of hyperalgesia, it is plausible to suggest that psychophysical stress prolongs inflammatory pain responses by augmenting intraganglionic ROS. Therefore, our data suggest ROS and TRPA1 interactions at the nociceptor level as another mechanistic link between psychophysical stress and inflammatory pain.

The level of ROS within sensory ganglia is dynamically regulated by oxidant and antioxidant enzymes under painful conditions, as nociceptor activities are highly susceptible to oxidative stress (53, 54). The overproduction of ROS under inflammatory and stressful conditions likely results from the net imbalance between the activities of multiple oxidative and antioxidative enzymes. However, despite the significant role of ROS in the pathogenesis of stress-induced exacerbation of inflammatory pain, the transcriptional profiles of genes associated with oxidative stress in sensory ganglia are poorly understood. The results from our unbiased oxidative stress PCR array of 94 genes under these conditions provided several novel findings. First, our data demonstrated that the combination of FSS with masseter inflammation led to the recruitment of additional genes exhibiting significant alterations in fold regulations compared to those observed under the sole inflammation condition throughout the progression of inflammation. Second, a higher count of genes exhibits noteworthy downregulation in comparison to those displaying significant upregulation, and the degree of fold regulation is more pronounced for the downregulated genes than for the upregulated ones. Third, inflammation and FSS are both characterized by altered expression of several key genes and gene families involved in oxidative stress, antioxidant defense, and reactive oxygen metabolism. These observations imply an altered expression pattern of oxidative stress genes, predominantly leaning towards downregulation as the production of ROS increases. This pattern may constitute a genetic compensation system that exerts protective metabolic effects to counteract the excessive production of ROS in a target tissue (55). Consequently, it appears that peripheral inflammation and psychophysical stress employ a shared set or sets of genes to intricately regulate ROS production in sensory ganglia, aiming to minimize the deleterious effects of oxidative damage on sensory neurons.

The gene most significantly downregulated is nicotinamide adenine dinucleotide phosphate (NADPH) oxidase 4 (*Nox4*), exhibiting a 21.5-fold reduction in expression seven days after CFA in stressed rats. The downregulation is more pronounced under stress conditions. The *Nox* enzyme family serves as the primary catalyst for oxidative stress, with the generation of ROS as their main function (56). *Nox4* is distinct from other *Nox* enzymes in that it generates predominantly H_2_O_2_ rather than O2^−^ due to its unique molecular structure (57). It contributes to chronic pain conditions through specific signaling mechanisms along nociceptive pathways (58). In rats with cancer-induced bone pain, *Nox4* expression significantly increases, and downregulating *Nox4* protein at the spinal cord level alleviates bone cancer pain (59) Nerve injury-induced mechanical hypersensitivity is significantly attenuated in mice with both global and inducible knockout of *Nox4* (60, 61). Pharmacological blockade of *Nox4* in the temporomandibular joint significantly attenuates synovial inflammatory responses (62). While these observations collectively suggest increased *Nox4* expression in the periphery and spinal cord as a causative factor in pathological pain conditions, the role of *Nox4* within sensory neurons remains unclear. Currently, the physiological consequences of the significant and consistent downregulation of *Nox4* in TG under masseter inflammation and stress conditions as we reported here can only be speculated upon. *Nox4* serves as a neuroprotective redox regulator for ROS and calcium homeostasis, preventing neuronal hyperexcitability and cell death (63). *Nox4* in skeletal muscle tissue facilitates ROS-mediated adaptive responses, promoting muscle function, maintaining redox balance, and preventing the development of insulin resistance (64). Therefore, our data suggest that inflammatory and stress conditions may compromise the protective role of *Nox4* by suppressing its expression, leading to hyperexcitability of TG neurons.

The *Hba1* gene encodes the alpha-globin protein of hemoglobin, which is the crucial protein in red blood cells responsible for transporting oxygen to cells and tissues throughout the body. *Hba1* is expressed not only in erythrocytes but also in non-erythrocytes, including neurons (65). To the best of our knowledge, there have been no reports on the expression of *Hba1* in sensory ganglia and its potential contribution to pain processing. Our findings revealed a consistent downregulation of *Hba1* expression in TG during inflammatory states, with an exacerbated effect under FSS. However, the exact impact of this downregulation on overall oxidative stress and inflammatory hyperalgesia remains unknown. Previous research has demonstrated that hemoglobin plays a protective role in hepatocytes, as oxidative stress leads to an upregulation of hemoglobin expression, and its overexpression suppresses oxidative stress (66). In light of this, our data suggest that the downregulation of *Hba1* during inflammation and stress may promote oxidative stress. Further investigation is warranted to elucidate the precise mechanisms and implications of *Hba1* modulation in the context of inflammatory responses and stress.

Another gene consistently downregulated, particularly under conditions of inflammation combined with stress, is glutathione peroxidase 3 (*Gpx3*). *Gpx*s represent a family of enzymes renowned for their role as major ROS scavengers, safeguarding cellular environments from the detrimental effects of excess ROS. Notably, *Gpx3* plays a crucial immunomodulatory role in cancer by regulating various pathways that counteract the effects of ROS (67) In a recent study, it was demonstrated that increased expression of *Gpx3* prevents tendinopathy in rats by effectively suppressing oxidative stress (65). These findings strongly imply that the consistent downregulation of *Gpx3*, especially when inflammation is coupled with stress, could play a pivotal role in altering the ROS balance. Consequently, therapeutic strategies aimed at increasing *Gpx3* expression in TG under these conditions may serve as a preventive measure against the exacerbation of pathological pain responses.

Among several genes exhibiting significant upregulation at multiple time points, dual oxidase 1 (*Duox1*) demonstrated the most pronounced increase. *Duox1* is a member of the NADPH oxidative enzyme family that generates ROS upon binding of calcium ions (68) Initially identified in the mammalian thyroid gland and referred to as thyroid oxidase (69), *Duox1* is expressed in various tissues, including the lung, placenta, liver, urothelial cells, and the brain (70, 71). Although *Duox1* expression has been identified in retinal ganglion cells (72), its presence in somatic ganglia has not been demonstrated. While the role of *Duox1* in pain processing has been rarely reported, a recent study revealed that *Duox1* expressed in keratinocytes contributes to nociceptive processing by modulating TRPA1 and redox-sensitive potassium channels in DRG sensory neurons as a paracrine mediator (73). Our study provides the first evidence of *Duox1* mRNA expression in TG. We have confirmed the presence of DUOX1 protein in TG, with its expression primarily localized to TG neurons. Our data suggest that *Duox1* may play a significant role in generating ROS under inflammatory and stress conditions. The temporal pattern of *Duox1* expression closely correlated with increased ROS levels under FSS, indicating that stress may enhance ROS generation in TG by upregulating *Duox1*. While the functional role of *Duox1* requires further investigation, our PCR array has identified novel genes or gene sets that could potentially be targeted for the management of pathological pain resulting from inflammation and psychophysical stress conditions.

In our current investigation, we have demonstrated that combining FSS with masseter inflammation results in a significant increase in ROS accumulation and *Trpa1* expression in the TG. Simultaneously, these changes coincide with the exacerbation of inflammatory hyperalgesia. Our PCR array data revealed that peripheral inflammation and psychophysical stress have distinct regulatory effects on the expression levels of various oxidative and anti-oxidative enzymes within TG, resulting in an imbalance that favors increased ROS levels within TG. The PCR array analysis of TG has provided valuable mechanistic insights, identifying novel genes or gene sets that could be potential targets for controlling oxidative stress within TG.

## Data Availability

The original contributions presented in the study are included in the article/Supplementary Material, further inquiries can be directed to the corresponding author.
